# Prediction of essential proteins based on gene expression programming

**DOI:** 10.1186/1471-2164-14-S4-S7

**Published:** 2013-10-01

**Authors:** Jiancheng Zhong, Jianxin Wang, Wei Peng, Zhen Zhang, Yi Pan

**Affiliations:** 1 School of Information Science and Engineering, Central South University, Changsha, Hunan 410083, PR China; 2 Department of Electronic and Information Engineering, Hunan Normal University, Changsha, Hunan 410083, PR China; 3 Department of Computer Science, Georgia State University, Atlanta, GA 30302-4110, USA

## Abstract

**Background:**

Essential proteins are indispensable for cell survive. Identifying essential proteins is very important for improving our understanding the way of a cell working. There are various types of features related to the essentiality of proteins. Many methods have been proposed to combine some of them to predict essential proteins. However, it is still a big challenge for designing an effective method to predict them by integrating different features, and explaining how these selected features decide the essentiality of protein. Gene expression programming (GEP) is a learning algorithm and what it learns specifically is about relationships between variables in sets of data and then builds models to explain these relationships.

**Results:**

In this work, we propose a GEP-based method to predict essential protein by combing some biological features and topological features. We carry out experiments on S. cerevisiae data. The experimental results show that the our method achieves better prediction performance than those methods using individual features. Moreover, our method outperforms some machine learning methods and performs as well as a method which is obtained by combining the outputs of eight machine learning methods.

**Conclusions:**

The accuracy of predicting essential proteins can been improved by using GEP method to combine some topological features and biological features.

## Background

Essential proteins are indispensable to support cellular life [1]. Identifying essential proteins can help us understand the minimal requirements for cell survival, which plays a significant role in the emerging field of synthetic biology [2]. Since the deleting, interrupting or blocking of essential proteins leads to the death of organisms, essential proteins can serve as candidates of drug-targets for developing novel therapies of diseases, such as cancers or infectious diseases [3]. Moreover, some studies have pointed out that essential proteins have correlation with human disease genes [4]. However it is expensive and time-consuming to experimentally identify essential proteins.

In recent years, many computational approaches have been presented to identify essential proteins according to their features. One group of researchers focus on detecting essential proteins based on their topological features in protein-protein interaction (PPI) networks, since previous studies have shown that the removal of those proteins with a larger number of neighbours in PPI networks is more likely to cause the organism to die [5]. Therefore, many centrality methods have been come up with such as Degree Centrality (DC) [6], Betweenness Centrality (BC) [7], Closeness Centrality (CC) [8], Subgraph Centrality (SC) [9], Eigenvector Centrality (EC) [10], Information Centrality (IC) [11], Edge Clustering Coefficient Centrality (NC) [12] and so on. However, these centrality methods have their own limits. For example, they highly depend on the accuracy of PPI networks and ignore the useful biological features. Recently, new methods that combine their topological features with their biological ones have been developed. Hart and his fellows have pointed out that the essentiality is a special property of protein complex and essential proteins are often highly clustered in certain protein complexes [13]. Based on this observation, Ren *et al. *[14] integrate PPI network topology and protein complexes information to predict essential proteins. According to the fact that proteins in protein complexes tend to be co-expressed, Li *et al. *[15] propose a new prediction method called PeC and Tang et al [16] propose another one, WDC, which integrates network topology with gene expression profiles. Considering the fact that essential proteins are more conserved than non-essential ones [17] and they frequently connects to each other [13], Peng *et al. *[15] have proposed an iterative method for the prediction of essential proteins based on the orthology and PPI networks. Their results show that the accuracy of predicting essential proteins can be improved by combing their biological features with their topological features. Although the methods mentioned above combine some features of essential proteins efficiently and explain how these features work together to decide the essentiality of proteins, better methods needs to be developed to integrate more appropriate features. Because there are different types of features that relates to protein essentiality, which suggests that multiple aspects of organisms contribute to determining the essentiality of proteins [18].

Another group of researchers use machine learning algorithms, such as support vector machine (SVM) [19], decision tree [20], Naive Bayes[21] and so on, to detect essential proteins. These methods train a classifier according to the features of known essential proteins and non-essential ones. Then test the classifier in the same organism or the other organisms. For example, Gustafson *et al. *[21] select a lot of essentiality related features including both topological features, such as the degree centrality (DC) in PPI networks, and biological features, such as open reading frame (ORF) length, Phyletic retention (PHY), paralogs, codon adaptation index (CAI) and so on. And then they use a Naive Bayes classifier to make prediction. Hwang *et al. *[19] build SVM classifier which combines the biological features, such as ORF length, strand and PHY, and topological features in PPI network, such as DC, BC, CC and so on. Additionally, some researchers combine several classifiers to predict essential proteins. Acencio *et al. *[20] learn 12 different network topological features (DC, BC, CC and so on) in the integrated network and some biological features, such as cellular localization and biological processes information. Then several decision tree-based classifiers are trained and tested based on these features. A best essentiality classifier is obtained by combining the outputs of these diverse classifiers. Deng *et al. *[22] also train their classifier by combining the results of four separate classifiers (Naive Bayes classifier, logistical regression model, C4.5 decision tree and CN2 rule). In [18], more approaches of detecting essential protein are introduced and discussed.

Now that those machine learning methods are available as software packages [23], they can be easily adapted to predict essential proteins using input features. However, it is difficult for them to explain how these features are used for classification. Moreover, few methods are able to automatically select appropriate features. Researchers tend to analyse the relationship between features and the essentiality of proteins with statistical methods. And then they decide which features are selected to train classifiers [19,22]. Gene expression programming (GEP) is a learning algorithm and what it learns specifically is about relationships between variables in sets of data, and it builds models to describe these relationships [24]. The features that show weak positive correlation with essentiality of proteins will not be selected by the GEP classifier.

In this work, we propose a GEP-based method to predict essential protein by combining biological features, such as subcellular location, and topological features, such as DC, BC, CC, SC, EC, IC, NC, and other composed features computed by the methods PeC, WDC and ION using biological and topological features. We carry out experiments on S. cerevisiae (Baker's yeast) data. The experimental results show that our method outperforms others using one of features calculated by existing methods (DC, BC, CC, SC, EC, IC, NC, PeC, WDC and ION). Moreover, in terms of area under an ROC curve (AUC), our method achieves better results than other machine learning methods (SVM, SMO, NaiveBayes, Bayes Network, RBF Network, J48, Random Tree, Random Forest, NaiveBayes Tree), and it performs as well as a method that uses multiple machine learning methods.

## Results and Discussion

In this section, we firstly analyze the results of 10-fold cross-validation. Then we compare the prediction of our method with other existing methods which calculate individual features. Moreover our methods are compared with other machine learning-based methods. Finally we show our best classifier and explain how to combine individual features to decide the essentiality of protein.

### Comparison of 10-fold cross-validation results

**Figure 1:**
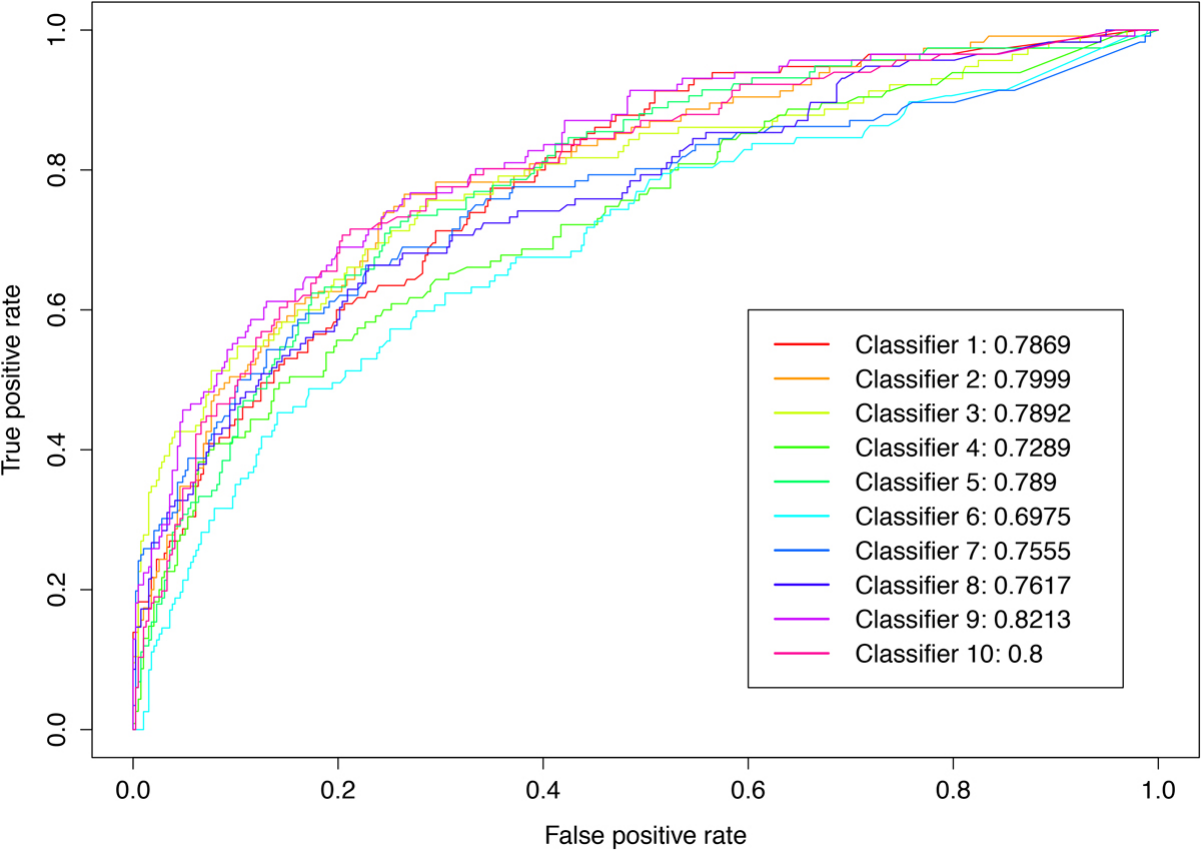
**ROC curves and AUC values of ten classifiers trained from 10-fold cross-validation**. Original data are divided into 10 equal datasets, and nine-folds are used to train the classifier and the remaining one fold is used for testing. The process is repeated ten times to generated ten classifiers, with each of the ten datasets used exactly once as testing data. The figure illustrates the ROC curves and corresponding AUC values of these classifiers.

There are ten classifiers from 10-fold cross-validation (see section Methods). The performance of each classifier is compared in terms of their ROC curves and the areas under the curves (AUC). As Figure 1 shown, the AUC values of these classifiers range from 0.6975 to 0.8213. The average AUC value is 0.7730. The performance fluctuation depends on the datasets. In this work, we select one with average AUC value among these classifiers to perform the following analyses.

### Comparison with other existing methods

**Figure 2:**
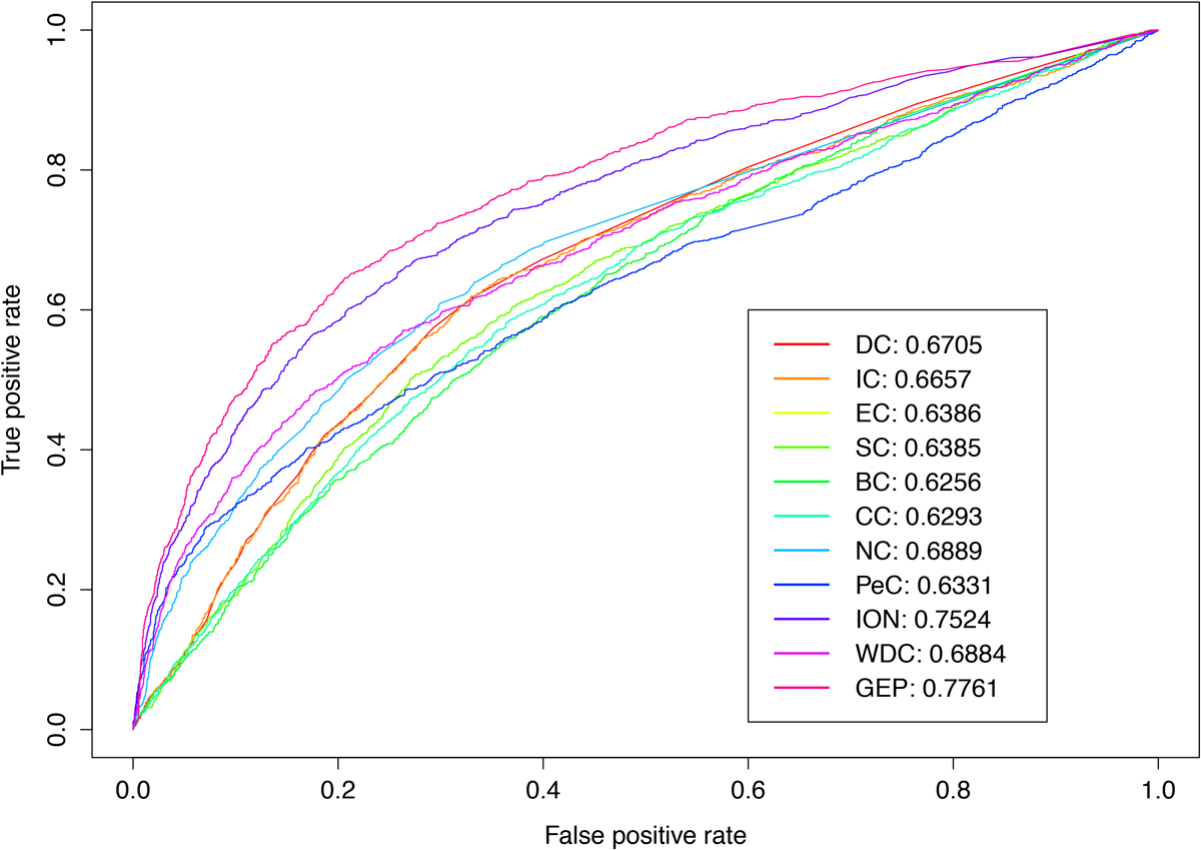
**ROC curves and AUC values of our GEP classifier and other methods using individual feature**. We select one classifier which has average prediction performance among ten classifiers generated from 10-fold cross-validation, and test it on original data containing 5093 proteins with all available learning features. The figure illustrates the ROC curves of our classifier and other methods that use individual feature.

Since our classifier combines the features calculated by other existing methods such as DC, BC, CC, EC, IC, SC, NC, PeC, WDC and ION, we compare its predictability with that of others. The proteins in PPI network are ranked in descend order according to the scores assigned by our classifier as well as these existing methods. After that the values of true positive rate (TPR) and false positive rate (FPR) are computed for each method with different top percentages of proteins selected as predicted essential proteins. The values of TPR and FPR are plotted in ROC curves with different cut-off values. As Figure 2 shown, the ROC curve of our classifier is obviously above that of the other existing methods. The AUC value of our GEP classifier is 0.7761 which is 0.0237 higher than ION which has the best performance among other existing methods, and is 0.113 higher than the average AUC value (= 0.6631) of these existing methods.

Specially, we select top 1167 proteins ranked by each method as predicted essential proteins. The rest of 3926 (= 5093-1167) proteins are regarded as non-essential ones. According to known essential protein, the values of sensitivity (SN), specificity (SP), positive predictive value (PPV), FPR, negative predictive value (NPV), F-Measure, accuracy (ACC) and Matthews Correlation Coefficent (MCC) are calculated for each method. Table 1 shows that the values of SN, SP, PPV, NPV, F-Measure, ACC and MCC of our GEP classifier are 0.5467, 0.8653, 0.5467, 0.8653, 0.5467, 0.7927 and 0.4120 respectively, which are higher than other existing methods. On the other hand, the FPR value of GEP is 0.1347, which is the lowest among all methods.

**Table 1:** Comparison between GEP and the methods using individual feature

Methods	SN	SP	FPR	PPV	NPV	F-measure	ACC	MCC
**GEP**	**0.5467**	**0.8653**	**0.1347**	**0.5467**	**0.8653**	**0.5467**	**0.7927**	**0.4120**
DC	0.4002	0.8217	0.1783	0.4002	0.8217	0.4002	0.7251	0.2219
BC	0.3505	0.8069	0.1931	0.3505	0.8069	0.3505	0.7023	0.1574
CC	0.3548	0.8082	0.1918	0.3548	0.8082	0.3548	0.7043	0.1630
SC	0.3676	0.8120	0.1880	0.3676	0.8120	0.3676	0.7102	0.1796
EC	0.3676	0.8120	0.1880	0.3676	0.8120	0.3676	0.7102	0.1796
IC	0.4010	0.8220	0.1780	0.4010	0.8220	0.4010	0.7255	0.2230
NC	0.4353	0.8321	0.1679	0.4353	0.8321	0.4353	0.7412	0.2674
PeC	0.4036	0.8227	0.1773	0.4036	0.8227	0.4036	0.7267	0.2263
ION	0.5124	0.8551	0.1449	0.5124	0.8551	0.5124	0.7766	0.3675
WDC	0.4576	0.8390	0.1610	0.4580	0.8388	0.4578	0.7516	0.2967

The proteins in PPI network are ranked in descend order according to the scores assigned by our classifier as well as these existing methods. we select top 1167 proteins ranked by each method as candidate essential proteins. The rest of 3926 (= 5093-1167) proteins are regarded as non-essential proteins. According to known essential protein, the values of sensitivity (SN), specificity (SP), positive predictive value (PPV), FPR, negative predictive value (NPV), F-Measure, accuracy (ACC) and Matthews Correlation Coefficent (MCC) are calculated for each method. The table lists the results.

### Comparison with other machine learning-based methods

To further evaluate the prediction performance of our classifier, we compare it with some machine learning methods, such as SVM, SMO, NaiveBayes, Bayes Network, RBF Network, J48, RandomTree, RandomForest, NaiveBayes Tree, Which are wildly used in previous prediction methods [19,21]. Acencio et al [19] build their classifier by combining eight decision tree classifiers (REP tree, naive bayes tree, random tree, random forest, J48, best-first decision tree, logistic model tree and alternating decision tree). We also compare our classifier with their composited classifier (named by Acencio). All of these machine learning based methods are implemented by using WEKA software package with default parameters setting and carried out 10-fold cross-validation based on topological features and biological features mentioned in section Method. Their average AUC values are listed in Table 2. As Table 2 shown, the average AUC value of GEP achieves 0.773, which outperforms all other machine learning methods. Compared to Acencio classifier, GEP classifier possesses almost equal prediction performance in term of their average AUC values.

**Table 2:** Comparison of average AUC between GEP and other machine learning based methods.

Methods	AUC
**GEP**	**0.773**
SVM	0.577
SMO	0.608
NaiveBayes	0.744
Bayes Network	0.731
RBF Network	0.669
J48	0.687
Random Tree	0.612
Random Forest	0.721
NaiveBayes Tree	0.746
Acencio	0.778

This table shows the average AUC values of our GEP classifiers and some machine learning methods.

### Analysis of GEP classifier

The GEP classifier produces an expression of different types of input features, which describes how the features are combined to decide the essentiality of protein. The expression of our GEP classifier with the best prediction performance is obtained as

$$\begin{array}{l} Min(lysosome,WDC-0.5)+(ION-0.9)+Max(\sqrt{0.4},Max(endoplasmicreticulum,\\ nucleus)-Min(0.5,SC))-Abs(Min(cytoplasm-vacuole,0.1),1)) \end{array}$$

As the expression shown, ION is a very predictive features. Because it relates to the evolutionary conservation of proteins, and essential proteins are often highly conserved across organisms [15]. The proteins located in endoplasmic reticulum or nucleus tend to possess indispensable functions, which is consistent with the observation of Acencio *et al. *[25]. However, those proteins located in cytoplasm or vacuole are less likely to be essential proteins. Note that some input features are not present in the expression. Our GEP classifier discards those features that are either less effective in predicting essentiality or replaced by other features. For example the classifier selects localized topological features such as WDC and SC instead of global ones such as BC, CC and IC. Because localized centrality measures can obtain better prediction performance than the global ones [26]. Both WDC and PeC depend on the features of co-expression and co-clustering of essential proteins, the classifier uses one of them in terms of their capability of prediction.

## Conclusions

As different types of features relates to the essentiality of proteins, it is still a big challenge for designing an effective method to predict them by integrating different features and explaining how these selected features decide the essentiality of protein. In this work, we propose a GEP-based prediction method which combines topological and biological features. Compared to other machine learning-based methods, it is able to select predictive features automatically and generates an expression that describes the relationships between them. We carry out experiments on S. cerevisiae (Baker's yeast) data.(1) Ten classifiers are obtained from 10-fold cross-validation based on all input features. The average AUC value is 0.7730. (2) In terms of average AUC values, our method outperforms a number of machine learning methods and has comparable performance to the method which combines the output results of eight decision trees. (3) We evaluate our classifier by testing it on all proteins in PPI network with all available learning features. The results indicate our classifier performs better than those that use individual features. Thus, our method can effectively combine a range of different features to predict essential proteins.

## Methods

### Experimental datasets

We implement experiments based on data of S. cerevisiae (Bakers' Yeast) because both its PPI and gene essentiality data are the most complete and reliable among various species. The PPI data of S. cerevisiae is downloaded from DIP database [27] using the version published on Oct.10, 2010, without self-interactions and repeated interactions. There are total of 5093 proteins and 24743 edges.

The list of essential proteins is integrated from the following databases: MIPS [28], SGD [29], DEG [1] and SGDP [30], which contains 1285 essential proteins. Among the 1285 essential protein, 1167 proteins present in PPI network. In our study, these 1167 proteins are regarded as essential proteins while other 3926(= 5093-1167) proteins are nonessential proteins.

The information of orthologous proteins used in method ION, is download from Version 7 of the In Paranoid database [31]. The gene expression data of yeast is retrieved from Tu *et al*., 2005 [32], containing 6,777 gene products (proteins) and 36 samples in total. Among the 6777 proteins, 4858 proteins are involved in the yeast PPI network. The subcellular information is downloaded from eSLDB database [33], which categorizes the 5093 proteins in PPI network into 16 different subcellular localizations.

### Features selection

Our GEP classifier is constructed to predict essential proteins based on topological and biological features. The topological features include degree centrality, betweenness centrality, closeness centrality, subgraph centrality, eigenvector centrality, information centrality and edge clustering coefficient centrality in PPI network, which are calculated by the centrality methods DC, BC, CC, SC, EC, IC and NC, respectively. Some composited features calculated by methods (PeC, WDC, and ION) which integrate the topological features with biological features are also used in this work.

Additionally, some biological features such as subcellular localization are considered. Because subcellular location plays a crucial role in protein function and proteins perform their functions in certain subcellular compartments. Acencio *et al. *[25] find that proteins located in nuclear subcellular compartments tend to be essential, because most essential biological processes for cell viability take place in nuclear. In contrast, most membrane proteins with functions as transporters or participate in metabolism related processes are more likely to be nonessential. In this work, all proteins in PPI network are involved in 16 different localizations including Vacuole, Vesicles, Lysosome, Membrane, Mitochondrion, Peroxisome, Secretory pathway, Cell wall, Cytoskeleton, Endoplasmic reticulum, Golgi, Transmembrane, Cytoplasm, Nucleus and Endosome, Extracellular.

For each of feature has its own value ranges, we normalize all features by dividing them by corresponding maximum values, so that ranges of features value are -1 to 1. The coefficient (0.1, 0.2, 0.5, and 1.0) is added to adjust the contribution of each feature.

### Classifier design

As one kind of the Evolutionary Algorithms (EA's), Gene expression programming (GEP) is a genotype/phenotype genetic algorithm that combines the merits of both genetic algorithms and genetic programming [24]. Each chromosome in GEP is expressed using nonlinear entities that can be represented as a fixed-length linear encoded string, such as mathematical expressions, polynomial constructs, logical expression, and so forth. Chromosomes can be evolved and new ones are generated by some genetic operations of mutation, transposition and recombination guided by a fitness function. GEP is able to do global searches for classification and performs well, but it is seldom adopted to solve the classification problem of essential proteins.

**Figure 3:**
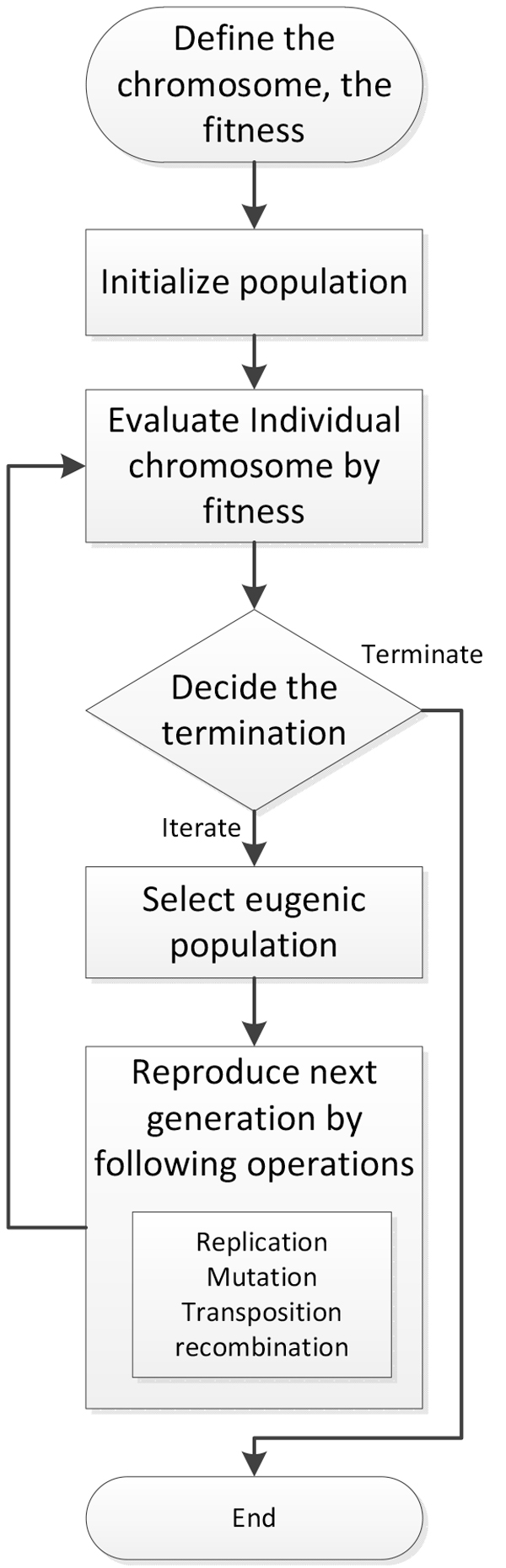
**Flowchart of building GEP classifier**. This figure shows the flowchart of building GEP classifier.

To build a classifier of predicting essential proteins using GEP the following major steps are needed: defining a chromosome using a function and terminal set, initializing a population and generating a group of chromosomes, defining a fitness function for evaluating chromosomes, selecting eugenic ones from populations, reproducing a group of chromosomes of the next generation, and deciding the termination of the model. Figure 3 illustrates the flowchart of building GEP classifier.

In this work, GEP is developed to predict essential proteins. First of all, a set of functions and terminals is chosen to define chromosomes that will be expressed as nonlinear entities. The set of functions contains arithmetic operators and logic operators, such as add, subtract, multiply, divide, min, max, equal, sqrt, log, exp, abs, while the set of terminals contains variables representing features of proteins (for example, topological and subcellular localization properties) and relevant coefficients. Then we build the chromosomal structure. For each chromosome the length of its head and the number of genes will be given.

The second step is to randomly generate first population. The input parameter indicates the size of population. We varied the number of populations from 800 to 12000, and kept track of results each model produces. According to the results, the performance of models increases gradually as the number of populations rises. Thus, we chose the maximum, 12000, as the number of populations.

The third step is to define a fitness function to evaluate individual chromosome. To obtain an optimal output, we select SSPN as our fitness function that is described as the product of sensitivity (SN), specificity (SP), positive predictive value (PPV), and negative predictive value (NPV). The formula is as follow:

(1) $$SSPN_{i}=S{N_{i}}^{*}S{P_{i}}^{*}PP{V_{i}}^{*}NPV_{i}$$

Where SN, SP, PPV, NPV are calculated respectively using following formulae for each chromosome i

(2) $$SN_{i}=\frac{TP_{i}}{TP_{i}+FN_{i}}$$

(3) $$SP_{i}=\frac{TN_{i}}{TN_{i}+FP_{i}}$$

(4) $$PPV_{i}=\frac{TP_{i}}{TP_{i}+FP_{i}}$$

(5) $$NPV_{i}=\frac{TN_{i}}{TN_{i}+FN_{i}}$$

Where TPi, TNi, FPi, and FNi indicate, respectively, the number of true positives, true negatives, false positives, and false negatives. Given protein features, we use the fitness function to compute scores for all chromosomes in population.

The forth step is to select the top 30% of populations as eugenic ones. Then the fifth step is that performing a set of genetic operations (including mutation, transposition and crossover) on eugenic ones reproduces chromosomes of the next generation that has the same size as former one.

Finally, we chose the maximum, 500, as the number of generations to decide the termination of the model. In this study, the parameters used in our GEP classifier are listed in Table 3. We develop the program that predicts essential protein based on GEP in C++ Language.

**Table 3:** Parameters used in our GEP method

Parameter	Description of parameter	Setting of parameter
P1	Number of Population	12000
P2	Length of Gene	1
P3	Length of Chromosome	60
P4	Length of Head	20
P5	Mutation rate%	0.25
P6	Cross rate%	0.1
P7	Number of Generation	500
P8	Function set	+,-,*, =,/, Sqrt, Log, Exp, Abs, Max, Min
P9	Fitness Function Name	SSPN

This table lists some parameters used in our GEP method.

### Training and testing set preparation

We build and evaluate our classifiers in terms of 10-fold cross-validation analysis, in which original data are divided into 10 equal datasets, and nine-folds are used to train the classifier and the remaining one fold is used for testing. Since the ratio of essential and non-essential proteins in original data is about 1:3.36 (essential proteins: non-essential proteins = 1167:3926), each fold data maintains the same ratio of essential proteins and nonessential proteins in original data. The cross-validation process is repeated ten times to generated ten classifiers, with each of the ten datasets used exactly once as testing data.

## Competing interests

The authors declare that they have no competing interests.

## Authors' contributions

JCZ and WP obtained the protein-protein interaction data, essential proteins, gene expression data and Orthologous data. JCZ and JXW designed the method. JCZ and WP analyzed the results. JCZ, WP, JXW discussed extensively about this study and drafted the manuscript together. ZZ and YP participated in revising the draft. All authors have read and approved the manuscript

## Declarations

The publication costs for this article were funded by the corresponding author.

This article has been published as part of *BMC Genomics *Volume 14 Supplement S4, 2013: Selected articles from the IEEE International Conference on Bioinformatics and Biomedicine 2012: Genomics. The full contents of the supplement are available online at http://www.biomedcentral.com/bmcgenomics/supplements/14/S4.
